# Body composition and risk of heart failure: protocol for a systematic review and meta-analysis

**DOI:** 10.1136/openhrt-2021-001632

**Published:** 2021-06-24

**Authors:** Ayodipupo S. Oguntade, Danyao Jin, Nazrul Islam, Reem Malouf, Hannah Taylor, Rishi Caleyachetty, Sarah Lewington, Ben Lacey

**Affiliations:** 1 Clinical Trial Service Unit & Epidemiological Studies Unit (CTSU), Nuffield Department of Population Health (NDPH), University of Oxford, Oxford, UK; 2 National Perinatal Epidemiological Unit, Nuffield Department of Population Health, University of Oxford, Oxford, UK; 3 MRC Population Health Research Unit, NDPH, University of Oxford, Oxford, UK; 4 UKM Medical Molecular Biology Institute (UMBI), Universiti Kebangsaan Malaysia, Kuala Lumpur, Malaysia

**Keywords:** heart failure, epidemiology, systematic reviews as topic, obesity, meta-analysis

## Abstract

**Introduction:**

Although there is strong evidence of an association between general adiposity and incidence of heart failure, previous systematic reviews and meta-analyses have not reliably assessed the association of heart failure risk with other aspects of body composition (such as body fat distribution or lean mass), or between body composition and risk of heart failure subtypes. We aim to conduct a systematic review and meta-analysis of prospective studies to address these uncertainties, and inform efforts to prevent and treat heart failure.

**Methods and analysis:**

The Preferred Reporting Items for Systematic Reviews and Meta-Analyses for Protocols statement was used as a template for this protocol. A systematic search of Medline, Embase and Global Health from database inception to present will be conducted to identify prospective studies reporting on the associations between major measures of body composition (body mass index, waist circumference, waist–hip ratio, total body fat, visceral adiposity tissue and lean mass) and risk of heart failure. Article screening and selection will be performed by two reviewers independently, and disagreements will be adjudicated by consensus or by a third reviewer. Data from eligible articles will be extracted, and article quality will be assessed using the Newcastle-Ottawa Scale. Relative risks (and 95% CIs) will be pooled in a fixed effect meta-analysis, if there is no prohibitive heterogeneity of studies as assessed using the Cochrane Q statistic and I^2^ statistic. Subgroup analyses will be by age, sex, ethnicity and heart failure subtypes. Publication bias in the meta-analysis will be assessed using Egger’s test and funnel plots.

**Ethics and dissemination:**

This work is secondary analyses on published data and ethical approval is not required. We plan to publish results in an open-access peer-reviewed journal, present it at international and national conferences, and share the findings on social media.

**PROSPERO registration number:**

CRD42020224584.

## Introduction

Heart failure (HF) is the pathophysiological endpoint of many cardiac diseases. There are about 65 million new cases of HF globally, and despite improvements in HF care over the last few decades, 5-year mortality continues to exceed 50% in most settings.^1 2^ HF is commonly classified into subtypes according to the proportion of ventricular blood volume remaining after systole (the ‘ejection fraction’), including HF with preserved ejection fraction (HFpEF), HF with reduced ejection fraction (HFrEF), and, more recently, HF with midrange ejection fraction (HFmEF).^3^ The clinical management of HF varies by HF subtype.

There is increasing evidence of the importance of adiposity, body fat distribution and lean (or ‘fat-free’) mass (collectively referred to here as ‘body composition’) to risk of cardiovascular disease.^4–8^ Body composition can be assessed directly using whole-body imaging (including dual energy X-ray absorptiometry, CT scans and MRI), or indirectly using bioelectric impedance or anthropometric measures.^7 9^ The relevance of the different measures of body composition to risk of HF is the subject of current investigation.^10–12^ The findings will be valuable in understanding the biological relationship between body composition and HF, and will inform efforts to prevent HF and manage patients with HF.

Body mass index (BMI; calculated by dividing weight in kilograms by the square of height in metres) is the most commonly used anthropometric measure of general adiposity. However, BMI is imprecise, it does not differentiate between body fat and lean mass and does not account for different distributions of body fat.^13 14^ The precise measurement and distinction between body fat distribution and lean mass are important in cardiovascular risk prediction in clinical practice. Therefore, the shortcomings of BMI have important implications for conditions such as HF, in which there can be rapid changes in body fat and lean mass. Although some studies have shown a linear relationship between HR risk and BMI, others have found a ‘J’-shaped association, with those in the underweight (BMI<18.5 kg/m^2^) and overweight or obese ranges (BMI>25 kg/m^2^) at higher risk than those in the normal range (18.5–25 kg/m^2^).^8 15–20^ For example, a recent study in the UK using the electronic health records of 2 million never smokers, found a J-shaped association of BMI with HF mortality with lowest risk in the 20–25 kg/m^2^; above this level, 10 kg/m^2^ higher BMI was associated with about 50% higher risk of HF mortality.^21^

The relationship between HF risk and measures of central adiposity, such as waist circumference or waist–hip ratio, has been less well studied than for BMI.^22 23^ However, some studies, such as the Health, Ageing and Body Composition study, have found waist circumference to be more strongly associated with HF risk than the BMI.^24^ Furthermore, prospective studies using whole-body imaging to directly measure body fat distribution have tended to be small, although there is some evidence of markedly strong associations of visceral adipose tissue with vascular risk factors, such as blood pressure.^25 26^ As such, the separate relevance of general and central adiposity to HF risk remains unclear, and the value of whole-body imaging above simpler anthropometric measures in the prognostic stratification of individuals in clinical practice is undetermined.^10 12^

In addition to general adiposity and body fat distribution, there is emerging evidence that lean body mass (measured using bioelectric impedance or whole-body imaging) is associated with cardiovascular risk.^27 28^ In particular, individuals with reduced lean mass (or ‘sarcopenia’) have been found to be at increased risk of HF, independent of level of adiposity.^29^ However, it remains unclear whether this is due to reverse causality, whereby HF causes loss of lean mass, or the effect of changes in the distribution of body fat, or reduced muscle mass itself.

The subtyping of HF based on left ventricular ejection fraction (LVEF) into those with preserved, midrange, or reduced ejection fraction, requires cardiac echocardiography or cardiac MRI. To date, few studies have achieved sufficient scale to reliably describe the association of body composition and HF ejection fraction subtypes. Obesity has been shown to be a risk factor for HFpEF in some observational studies but data on HFrEF and HFmEF are scarce.^22 30–33^ Furthermore, previous reviews have tended not to assess the effect of body fat distribution on risk of different HF LVEF subtypes, or on classifications of HF based on likely aetiology, such as ischaemic and non-ischaemic HF.^8 19^ This is especially important since the association between body composition and HF varies by HF aetiological types as shown in recent studies in the Swedish population.^34 35^

In summary, although there is strong evidence of an association of obesity (as measured by BMI) with incidence and mortality from HF, previous systematic reviews and meta-analyses have not reliably assessed the association of HF risk with body fat distribution and lean mass, or between any of the measure of body composition and risk of HF LVEF subtypes or HF aetiologies. This is in part because such studies require measures of bioelectric impedance, whole-body imaging or measures of cardiac function that it has not been feasible to include in large-scale studies until recently. We aim to conduct a systematic review and meta-analysis of prospective studies to address these uncertainties, and inform efforts to prevent and treat HF.

### Objectives

The primary objective of this study is to determine the associations of HF incidence with body composition. The secondary objective is to determine the extent to which these associations vary by age, sex, ethnicity, HF LVEF subtypes and HF aetiologies.

### Review questions

1. What is the association between general adiposity (as measured by BMI and total body fat) and HF incidence?
2. What is the association between central adiposity (as measured by waist circumference, waist–hip ratio and visceral adipose tissue) and HF incidence?
3. What is the relationship between lean body mass and HF incidence?
4. To what extent do these associations between body composition and HF incidence vary by age, sex, ethnicity, HF LVEF subtypes and HF aetiologies?

## Methods and analysis

This study will be a systematic review and meta-analysis of prospective studies which report the association between body composition measures and HF risk. We followed the Preferred Reporting Items for Systematic Reviews and Meta-Analyses-Protocols (PRISMA-P) review guidelines to draft our protocol (see PRISMA-P checklist in online supplemental appendix 1).^36^ This is a standardised format for reporting systematic review protocol that guarantees accuracy, completeness and transparency of systematic review protocols. This review is registered on PROSPERO (number CRD42020224584), which is an international prospective register of systematic reviews.^37^ Registration of the protocol in a prospective register prevents duplicity of studies and strengthen the rigour of the proposed study and compliance with systematic review standards.

10.1136/openhrt-2021-001632.supp1 Supplementary data

### Eligibility criteria

Eligibility criteria will be defined using the participants, exposures, outcomes and study type strategy.^38^ Studies that meet the following criteria will be included in the review: (1) participants must be 18 years or over; (2) population-based prospective studies reporting the association between body composition (BMI, waist circumference, waist–hip ratio, total body fat, visceral adiposity tissue, and lean mass) and HF incidence; (3) studies must report outcomes as incident HF diagnosis, HF hospitalisation or HF mortality; (4) study types eligible for inclusion will be prospective cohort, nested case-control, retrospective cohort or randomised controlled trials; (5) studies must have been published in English.

### Exclusion criteria

We will exclude: (1) studies other than prospective studies, such as case-control studies (except nested case-control studies), review articles, conference abstracts, editorials, letters to the editor and cross-sectional studies; (2) studies which do not report effect sizes of associations between the selected measures of body composition and HF incidence; (3) studies conducted in participants with established HF; (4) studies with incomplete data (which preclude reliable analysis) which could not be obtained after reasonable request from the corresponding author of such studies; (5) studies with too few HF events for reliable analyses (defined here as 20 events or fewer).

### Search strategy

We will conduct a systematic search of Medline, Embase and Global Health databases for articles published from the inception of each of the databases to present using the search strategy developed in conjunction with the information specialist (NR). The MeSH terms were chosen from the thesaurus used for indexing the subject headings. We also checked the search strategy used in previous systematic reviews.^8 19^ MeSH search terms and textwords associated with ‘body composition’, ‘adiposity’, ‘lean mass’, ‘obesity’, ‘sarcopenia’, ‘heart failure’, ‘cardiac dysfunction’, ‘ventricular dysfunction’, ‘cardiomyopathies’, ‘cohort studies’ and ‘adults’ will be used for the searches. Reference lists of eligible articles will also be screened for inclusion. Full details of the search strategy in Medline are shown in table 1 below. The syntax of this search strategy will be adapted to Embase and Global Health databases. The search will be limited to English, adults and observational studies or randomised controlled trials with no restriction by date.

**Table 1:** Search strategy in Medline

Search	Keywords	Thesaurus (MeSH)	Textwords
#1	Exposure	Body Composition/ Body Weights and Measures/ Adipose Tissue/ Obesity/ Obesity Hypoventilation Syndrome/ Obesity, Abdominal/ Obesity, Metabolically Benign/ Obesity, Morbid/ Obesity Management/ Bariatrics/ Metabolic Syndrome/ Adipocytes/ Adiposity/ Body Fat Distribution/ Anthropometry/ exp Subcutaneous Fat/ exp Subcutaneous Fat, Abdominal/ Body Mass Index/ Body Weight/ Body Height/ Waist Circumference/ Waist-Height Ratio/ Waist-Hip Ratio/ Body Constitution/ Somatotypes/ Body Size/ Overweight/ Abdominal Fat/ Body Weight Changes/ Sarcopenia/ Thinness/ Cachexia/ Intra-Abdominal Fat/	((body or abdom* or intraabdom* or central or truncal or trunk or appendicular or subcutaneous or sub-cutaneous or visceral or limb or arm or leg or peripheral or android or gynoid) adj fat?).mp body composition* body weight and measure* adipos* obes* metabolic syndrome* overweight* BMI* adipocyt* fat distribution* fat mass* anthropometr* quetelet* index* body weight* body height* waist circumference* waist-height ratio* hip circumference* waist-hip ratio* body constitution* Somatotypes* body size* body mass* sarcop?enia* thinness* muscle mass* muscle bulk* lean mass* fat-free mass* skeletal bulk*
#2	Outcomes	exp Heart Failure/ Pulmonary Edema/ Ventricular Dysfunction/ Ventricular Dysfunction, Left/ Ventricular Dysfunction, Right/ Cardiomyopathy, Dilated/ exp Cardiomyopathies/ Cardiomegaly/ Hypertrophy, Left Ventricular/ Hypertrophy, Right Ventricular/ exp Ventricular Function/	heart failure* cardiac failure* diastolic HF* systolic HF* pulmonary o?dema HFrEF* HFpEF* HFmEF* ventricular failure* biventricular failure* cardiac dysfunction* ventricular dysfunction* cardiomyopath* dilated cardiomyopath* cardiorenal syndrome* cardiomegaly* ventricular* hypertrophy* cardia* hypertrophy* ventricular function ventricular remodeling* cardia* remodeling* BNP* NT-BNP* natriuretic peptide*
#3	Study type	exp Cohort Studies/ Observational Study/	cohort* longitudinal* prospective* follow-up* observational * incidence stud*
#4		#1 AND #2 AND #3	

### Data management and article screening

Abstracts from articles identified from the database searches will be imported into EndNote reference manager to allow duplicate citations to be deleted. The remaining abstracts will then be uploaded to ‘Covidence systematic review’ a webpage portal which allows collaborations between two or more reviewers for article screening and selection, based on the study inclusion and exclusion criteria.^39^ Title and abstract screening will be undertaken by two review authors (ASO and DJ), blinded to each other’s selection. Disagreements will be resolved by discussion and consensus between the two reviewers using the full text version of the selected articles, or where necessary a third reviewer. Where multiple publications are available from a single cohort study, the article to report on the greatest number of HF events will be included. A flow chart of the study selection process will be presented in accordance with the PRISMA statement.^40^

### Data extraction and management

Data will be collected from eligible articles using predesigned data extraction form. We will develop and pilot-test the data collection form before the start of the study and extract the following variables from each of the eligible articles: name of first author, year of publication, country of study, year of baseline survey, mean follow-up time, selection criteria for study participants, baseline characteristics (including number of participants, mean age and per cent women), measures of body composition at baseline, date of resurvey (if performed), number of new cases of HF (fatal and non-fatal), LVEF among patients with HF, HF subtype(s), HF aetiologies, details of statistical analyses performed (including exclusions, type of statistical model, confounders, crude and adjusted relative risk (RR) estimates and 95% CIs).

### Assessment of study quality and risk of bias

Quality of included studies will be assessed using the Newcastle-Ottawa Scale (NOS) which has been validated for use in systematic reviews.^41^ This will be done by two review authors (ASO and DJ), blinded to each other’s selection. Disagreement between the review authors will be resolved by a panel review made up of other study reviewers (NI, BL and RM). The NOS uses three quality assessment parameters (study group selection, group comparability and outcome assessment).^42^ These quality metrics are divided into an eight-item list using a point score system. A score of 9 points denotes studies of highest quality while less than 5 points denotes high risk of bias.^41 42^

### Data synthesis and analysis

Descriptive characteristics of included studies will be reported, together with tables of the RRs extracted from each study. For the systematic review, findings from each study will be discussed in qualitative analyses. For meta-analyses, the RR estimates and 95% CIs for the association between different body composition measures and HF events will be extracted from each study and pooled using a fixed effects model. We will abstract the risk estimates from the most fully adjusted model except where such models adjusted for potential intermediate risk factors in a further step. In such cases, we will use models which did not include such intermediate factors. Where studies only reported RR in subgroups, we will use fixed-effect meta-analysis to generate an overall study-level RR so that each study will be represented only once in the main analysis. Where possible, subgroup analyses will be performed for each body composition measure and HF outcome, by age, sex, ethnicity, HF LVEF subtypes and HF aetiologies (eg, ischaemic heart disease, cardiomyopathy, congenital heart disease, valvular heart disease, hypertension or diabetes). Risk estimates and their 95% CI will be rescaled to 1-SD increase where appropriate to facilitate comparison across studies. Sensitivity analysis will be performed by restricting analyses to studies with a low risk of bias. In sensitivity analyses, we will test for the effect of (1) including studies which reported on HF aetiologies (eg, ischaemic heart disease, cardiomyopathy, congenital heart disease, valvular heart disease, hypertension or diabetes) and HF LVEF subtypes (HFpEF, HFrEF and HFmEF); (2) excluding studies which used national databases as opposed to prospective cohort analysis; (3) excluding individual studies to investigate extreme results. Meta-analyses of reported associations will be presented using forest plots. Heterogeneity between studies will be determined using Q statistic and I^2^ statistic. We will consider I^2^>50% as a significant level of heterogeneity.^43^ Publication bias will be assessed using funnel plots and Egger’s test. The quality and risk of bias of included studies will also be presented in tables and discussed in the final publication. Data analysis will be conducted using SAS software, V.9 (SAS Institute).

## Conclusion

This systematic review will provide answers on the biological relationship between different body composition indices and incident HF risk. This will be invaluable for risk prediction of different HF types in clinical practice. It will also shed light on the independent and additive role of whole-body imaging to conventional anthropometric indices in the risk stratification of patients in primary care. This is especially important given the increased prevalence of overweight and obesity globally in the adult workforce.^44^ This will contribute to the evidence base for weight management and weight reduction strategies in reducing HF risk.

## Data Availability

Data sharing is not applicable as no datasets generated and/or analysed for this study.
